# 

**Published:** 1814-10

**Authors:** 


### MONTHLY CATALOGUE OF MEDICAL BOOKS.

A VIEW of the Comparative Advantages and Disadvantages of the Navy and Army Surgeon, and of the Surgeon in private Practice; together with a proposed Amendment of the Condition of Assistant-Surgeons at their outset in the Navy. By William Cullen Brown, M. D. Ed. Surgeon of His Majesty's prison-ship Arve Princen. 1814.

The Accoucheur's Vade-Mecum; being the Substance of a series of Lectures delivered at the Westminster Lying-in Institution, Queen-square. Second edition, revised and corrected. 12mo. boards, 7s.

Thoughts on Puerperal Fever, and its Cure by Spirits of Turpentine; illustrated by Cases in the Lying-in Hospital, Dublin, &c. By John Brenan, M.D. 8vo. sewed, 1s.

Observations on the Nature and Cure of Dropsies, and particularly on the presence of the Coagulable part of the Blood in Dropsical Urine; to which is added, an Appendix, containing several Cases of Angina Pectoris, with Dissections, &c. The second edition, corrected and improved. By John Blackhall, M.D. Physician to the Devon and Exeter Hospital, and to the Lunatic Asylum near Exeter. 8vo. boards, 12s.

A Catalogue of an extensive Collection of new and second-hand Medical Books; to which is added, a complete List of the Lectures delivered in London, with their terms, hours of attendance, &c. and Tables of the Pay of the Medical Department of the Army, Navy, and East India Company's service. By John Anderson (late Underwood,) 4, West Smithfield.

### NOTICES TO CORRESPONDENTS.

*We regret exceedingly to have been under the necessity of postponing Dr. Sutton's answer to Mr. Want, but our original department was completed before it came to hand.*

*Upon the subject of Dr. Sutton's second letter, and the anonymous enclosure, Mr. Want assures Dr. S. that the writer of it is completely mistaken as to the circumstances there spoken of: the letter will, however, be published in our next Number, accompanied with a statement of facts relative to the domestic occurrence to which he refers, of which an account was not only given to the Medical Society, but has been and is still related in the most public manner to most individuals with whom Mr. Want converses upon the subject, and most especially to the gentlemen attending the Meetings of Sir J. Banks.*

